# Late-afternoon endurance exercise is more effective than morning endurance exercise at improving 24-h glucose and blood lipid levels

**DOI:** 10.3389/fendo.2022.957239

**Published:** 2022-07-19

**Authors:** Hyeon-Ki Kim, Shota Furuhashi, Masaki Takahashi, Hanako Chijiki, Takuya Nanba, Takayuki Inami, Zsolt Radak, Shizuo Sakamoto, Shigenobu Shibata

**Affiliations:** ^1^ Faculty of Science and Engineering, Waseda University, Tokyo, Japan; ^2^ Institute of Physical Education, Keio University, Yokohama, Japan; ^3^ Graduate School of Advanced Science and Engineering, Waseda University, Tokyo, Japan; ^4^ Institute for Liberal Arts, Tokyo Institute of Technology, Tokyo, Japan; ^5^ Faculty of Sport Sciences, Waseda University, Saitama, Japan; ^6^ Research Center for Molecular Exercise Science, University of Physical Education, Budapest, Hungary; ^7^ Faculty of Sport Sciences, Surugadai University, Saitama, Japan

**Keywords:** chrono-exercise, exercise timing, endurance exercise, glucose levels, triglycerides

## Abstract

**Background:**

Glucose and lipid tolerance reportedly exhibit diurnal variations, being lower in the evening than in the morning. Therefore, the effects of exercise on glucose and blood lipid levels at different times of the day may differ. This study aimed to investigate the effects of short-term endurance exercise intervention in the morning versus late afternoon on 24-h blood glucose variability and blood lipid levels.

**Methods:**

Twelve healthy young men participated in a randomized crossover trial. The participants were assigned to morning (09:00–11:00) or late afternoon (16:00–18:00) endurance exercise for a week, consisting of supervised exercise sessions on Mondays, Wednesdays, and Fridays. In the morning and evening trials, the participants walked for 60 min on a treadmill at approximately 60% of maximal oxygen uptake (VO_2max_). Following a 2-week wash-out period, the participants performed the exercise training regimen at another time point. Continuous glucose monitoring was used to evaluate blood glucose fluctuations during each 24-h trial period. Blood samples were collected before and after each intervention to examine blood lipid and hormonal responses.

**Results:**

Examination of the area under the curve (AUC) of the glucose level changes for 24 h after the late afternoon versus morning exercise intervention revealed significantly lower values for the former versus the latter (*P* < 0.01). The AUC of glucose level changes after each meal was also lower after the late afternoon versus morning intervention, and significantly lower values were observed in the late afternoon versus morning trial for breakfast and dinner (*P* < 0.05, *P* < 0.01). In addition, a significant decrease in triglycerides (TG) and TG/high-density lipoprotein cholesterol (HDL-C) was noted after versus before the late afternoon intervention (*P* < 0.05).

**Conclusions:**

These results suggest that late afternoon endurance exercise is more effective than morning endurance exercise at improving 24-h glucose and triglyceride levels.

## Introduction

The World Health Organization reported that an estimated 17.9 million people died from cardiovascular diseases (CVDs) in 2019, representing 32% of all global deaths. CVD is the leading cause of death globally (1). Hyperlipidemia and hyperglycemia are also independent risk factors of CVDs (2, 3). Type 2 diabetes and obesity are related to increased blood lipid and glucose levels (4, 5). Therefore, the prevention and improvement of hyperlipidemia and hyperglycemia are important for reducing the risk of CVDs.

Circadian rhythms in mammals are associated with the regulation of several physiological and biological functions, including body temperature, sleep-wake cycles, physical activity, hormone secretion, and metabolism, which are controlled by clock genes. This clock system collectively regulates a wide range of metabolic targets, including glucocorticoids (6), the master energy sensor adenosine monophosphate-activated protein kinase (AMPK) (7), the rate-limiting step in fatty acid and cholesterol synthesis (8, 9), and the hepatic cAMP response element-binding protein, which regulates gluconeogenesis (10). As a result, various metabolic processes, such as insulin sensitivity, insulin secretion, cholesterol synthesis, fat oxidation, and energy expenditure, are in rhythm throughout the 24-h daily cycle (11–13). In addition, the disruption of circadian rhythms is associated with the development of metabolic diseases such as obesity and diabetes. Therefore, the timing of exercise and nutritional intake should be considered accordingly to reduce the risk of obesity, diabetes, and other metabolic diseases.

It is well known that endurance exercise has a favorable effect on many established CVD-related risk factors (14, 15). Hence, exercise guidelines mention the exercise modality, intensity, duration, and frequency to prevent and improve hyperlipidemia and hyperglycemia (14, 15). However, no mention has been made regarding exercise timing. One reason for this is that the effects of exercise timing on organisms have not been adequately examined. However, glucose and lipid tolerance reportedly have diurnal variations, being lower in the evening than in the morning (16–18). Previous studies demonstrated that diurnal variations in glucose tolerance are so great that adults with normal glucose tolerance in the morning are metabolically equivalent to prediabetics in the evening (17, 19). In addition, postprandial triglyceride concentrations were higher in the evening than in the morning, possibly because of diurnal variations in lipid tolerance (20, 21). Therefore, different exercise timings exert different effects on glucose and blood lipid levels. Previous studies indicated that lipolysis and fat oxidation are higher in the evening versus morning acute endurance exercise (22, 23).

However, the effects of exercise at different times of day (e.g., morning versus late afternoon) on glucose levels and lipid markers have not been clarified. Thus, this study aimed to compare the effects of short-term endurance exercise performed in the morning versus late afternoon on glucose and blood lipid levels in healthy young men.

## Methods

### Participants

**Figure 1:**
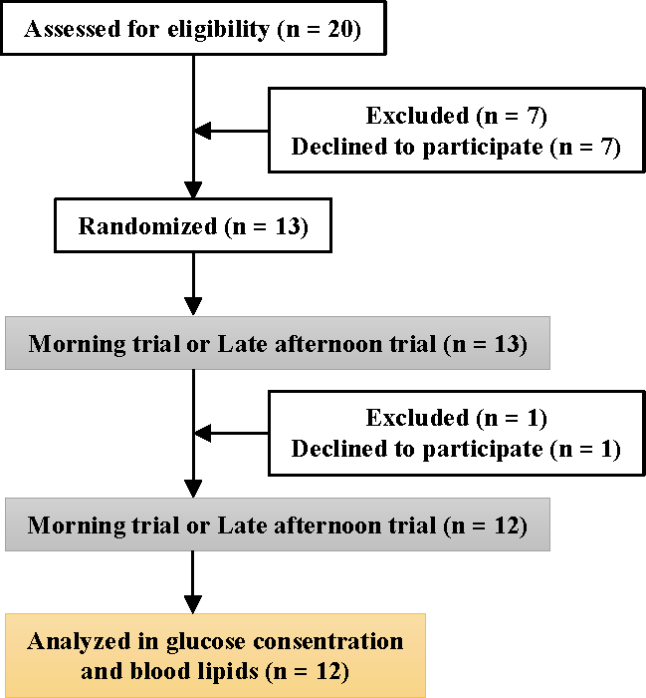
Consolidated standards of reporting trials flow diagram.

Twelve healthy young men aged ≥20 years (21.8 ± 0.2 years, mean ± SE) without regular exercise habits participated in the present study after providing informed consent. The participants were recruited only if they met the following inclusion criteria (1): no anti-obesity or anti-diabetic supplement use (2); no medical diagnosis of diabetes or dyslipidemia (3); no hypertension (systolic blood pressure >140 mmHg, diastolic blood pressure >90 mmHg); and (4) no use of blood glucose/lipid-lowering or related medications. Therefore, the study included only participants with no medication use, disease history, or smoking habits at the initial recruitment stage (**Figure 1**).

This study was conducted in Tokyo (Japan) between August 2019 and February 2021. The participants made no special lifestyle changes during the study period. This study was approved by the Ethics Committee of Waseda University (approval no. 2018-136 (1)) and conducted in accordance with the Declaration of Helsinki guidelines. The human trial in the present study was registered at https://center6.umin.ac.jp/cgi-bin/ctr/ctr_view_reg.cgi?recptno=R000042147 as UMIN000036994 .

### Preliminary measurements

Height, body weight, and body fat percentage were measured at the participants’ first visit to the laboratory. All participants underwent an incremental exercise test to exhaustion on a treadmill to determine the maximal oxygen uptake (VO_2max_). The incremental exercise test used a treadmill (MAT-2700; Fukuda Denshi, Tokyo, Japan) and the Bruce protocol, in which the incline and speed were increased every 3 min (24). The results of the incremental exercise test were used to calculate the 60% VO_2max_ value for each participant.

### Endurance exercise intervention

**Figure 2:**
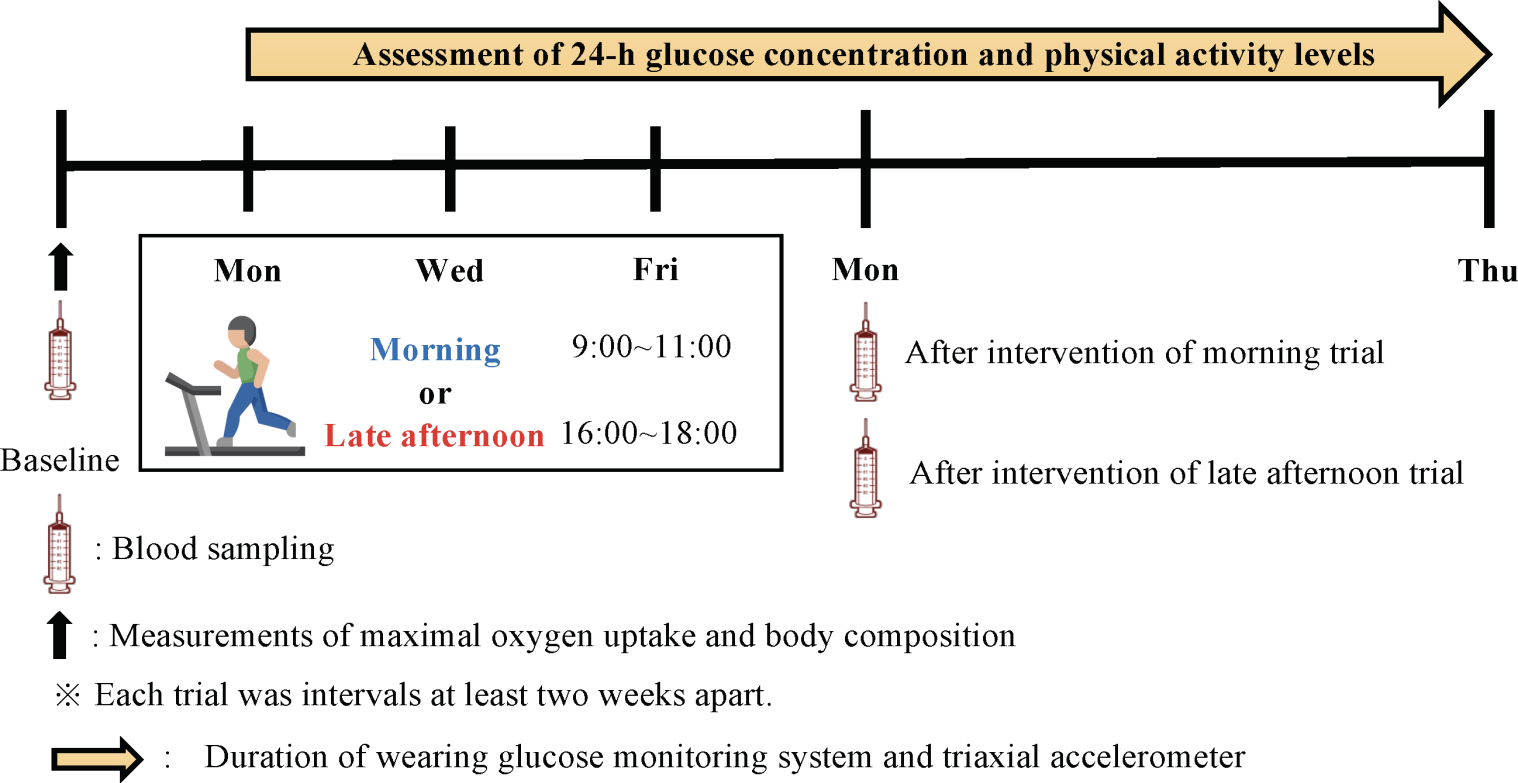
Experimental design of the study.

A randomized crossover design was used in this study. Participants were assigned to either morning (9:00–11:00) or late afternoon (16:00–18:00) endurance exercise training for a week, which consisted of supervised exercise sessions on Mondays, Wednesdays, and Fridays. Exercise intensity was changed by adjusting the speed of the treadmill 5–10 min after the start of loading. Heart rate was measured using a heart rate sensor to align the exercise intensity for each exercise trial. (Polar H10; Polar, Tokyo, Japan). In the morning and late afternoon trials, the participants walked for 60 min on a treadmill at approximately 60% VO_2max_. Following a 2-week wash-out period, the participants performed the exercise training regimen in another trial (**Figure 2**). All participants were instructed to follow the same daily routine with a similar diet as much as possible during both trial periods.

### Anthropometry

Body weight was measured to the nearest 0.1 kg using a digital balance (Inbody 270; Inbody Inc., Tokyo, Japan), and height was measured to the nearest 0.1 cm using a wall-mounted stadiometer (YS-OA; As One Corp., Tokyo, Japan). Body mass index was calculated as the weight in kilograms divided by the square of height in meters. Body fat Percentage was measured *via* direct segmental multi-frequency bioimpedance analysis (InBody270; Inbody Inc., Tokyo, Japan).

### Measurement of physical activity

All participants were asked to wear a triaxial accelerometer (Active style Pro HJA-750C; Omron Corp., Kyoto, Japan) during the trial period. They wore the triaxial accelerometer each day at all times from morning to night except during showers and sleeping hours. We selected those wearers who wore the device for at least 600 min and averaged the data to calculate daily physical activity. We used moderate-to-vigorous physical activity (MVPA) and step counts required for the assessment (25).

### Measurement of dietary and chronotype

To determine the chronotype of each participant (morningness to eveningness), a lifestyle survey was conducted using the Morningness-Eveningness Questionnaire (26). The MEQ has consisted of 19 questions related to sleep habits, sleepiness, and the preferred time for daily performance. The scores ranged from 16 to 86. Based on their scores, the participants were divided into the following three chronotype groups: morningness (score 59–86), intermediate (score 42–58), or eveningness (score 16–41). The Food Frequency Questionnaire was used to assess the participants’ dietary and nutritional intake. Most FFQs for Japanese people are highly effective for estimating nutrients (27). The FFQ asks about the frequency of food intake (once daily, once or twice a week, once or twice a month, etc.) and the approximate serving size (weight, volume, size). The average daily energy intake was depicted as kilocalories per day (kcal/day). The participants were asked to record the timing of each meal intake during each trial period (**Table S1**).

### Measurement and analysis of glucose levels

All participants were asked to wear a continuous glucose monitoring system (FreeStyle Libre Pro; Abbott Laboratories, Chicago, IL, US) to collect the continuous measurement of glucose levels during the entire trial. Once applied, the system can continuously measure glucose levels in 15 min intervals for 14 days. The sensor was applied to the back of the upper arm. The sensor continually stores data on glucose levels. In both trials, 24-h glucose levels and area under the curve (AUC) were calculated. The 24-h glucose variability for each exercise trial was examined from Tuesday through Friday. The 24-h glucose variability from Saturday after each intervention to Thursday of the following week was also examined. The 24-h glucose variability measurements were taken from 00:00 h to 24:00 h on that day. Postprandial glucose fluctuations were examined up to 4-h postprandial based on each meal time that was recorded. The parameters used to determine glycemic variability included mean glucose level (MAX), minimum glucose level (MIN), mean amplitude of glycemic excursion (MAGE), and J-index. The J-index was calculated using the following formula (28): J-index = 0.001 × (mean ± standard deviation)^2^.

#### Measurement of blood glucose and lipid levels

Venous blood samples were collected at baseline and after each trial (**Figure 2**). The participants were required to abstain from any intense exercise for at least one day before the collection of the blood sample and fast for at least 10 h overnight. The blood samples were collected at 09:00–10:00, and blood samples after each trial intervention were drawn at least 48 h after the end of the last trial. Blood samples were collected in a tube containing thrombin and a heparin-neutralizing agent, and a tube containing ethylenediaminetetraacetic acid (EDTA)-Na_2_. After being collected, the blood intended for serum analysis was allowed to stand for 30 min at room temperature, whereas the blood intended for plasma analysis was centrifuged at 3500 rpm for 10 min. After centrifugation, the serum and plasma samples were extracted from the respective blood collection tubes and stored at −80°C until the assay. Plasma insulin, glucose, and serum blood lipids (triglycerides [TG]; high-density lipoprotein cholesterol [HDL-C]; low-density lipoprotein cholesterol [LDL-C]; apolipoprotein A1 [ApoA1]; apolipoprotein B [ApoB]) were analyzed by Kotobiken Medical Laboratories Inc. (Tokyo, Japan). Cholesterol uptake capacity (CUC) was analyzed by Sysmex Corporation (Hyogo, Japan).

### Statistical analysis

Predictive Analytics Software for Windows (SPSS Japan Inc. Tokyo, Japan) was used for the data analysis. The total sample size was calculated to detect the medium effects. Using a medium effect size (f = 0.25), a sample size of 12 participants was deemed sufficient to provide 80% power to detect (α-error probability value set at 0.05) within–between interactions (two trials: morning and evening trials) using measures taken at morning and late afternoon trials time points (G*Power, version 3.1.9.2; Universitat Kiel, Germany). The normal or non-normal distributions of the data were analyzed using the Shapiro–Wilk test. To compare changes in the diurnal and postprandial glucose levels between trials, a two-way repeated-measures analysis of variance (ANOVA) was used (effects of trial and time were used as factors). Wilcoxon’s signed-rank test was used for the 24-h AUC per day of the intervention period for each trial, for which normality and equal variances were not assumed. Repeated one-way ANOVA was also performed to compare blood parameters at baseline versus after the intervention for each trial. The Friedman test was used for TG and TG/HDL-C, for which normality and equal variances were not assumed. In addition, a paired t-test was used to compare glucose parameters and AUC during the intervention period in both trials. Statistical significance was set at *P* < 0.05.

## Results

### Characteristics of study participants

Participant characteristics are shown in **Table 1**.

**Table 1:** Characteristics of study participants.

	All participants (n = 12)
**Age (years)**	21.8 ± 0.2
**Height (cm)**	173.0 ± 1.3
**Body Mass (kg)**	64.2 ± 2.8
**Body mass index (kg/m^2^)**	21.3 ± 0.9
**%Fat (%)**	16.8 ± 1.1
**VO_2max_ (ml/min/kg)**	45.9 ± 2.0
**Morningness-Eveningness Questionnaire (score)**	63.2 ± 1.1
**Energy Intake (kcal/day)**	2100.2 ± 102.3

All data are presented as mean ± standard error, BMI: body mass index, %Fat: body fat percentage, VO_2max_: maximal oxygen uptake.

### Comparison of physical activity levels and mealtime

There were no significant inter-trial differences in the amount of physical activity (step count and MVPA) or mealtime during any trial period (**Table S1**).

### Comparison of the heart rate during the exercise in each trial

There was no difference in heart rate between the morning and late afternoon trials during exercise (**Figure S1**).

### Comparison of diurnal changes in glucose concentrations and AUC of each trial

**Figure 3:**
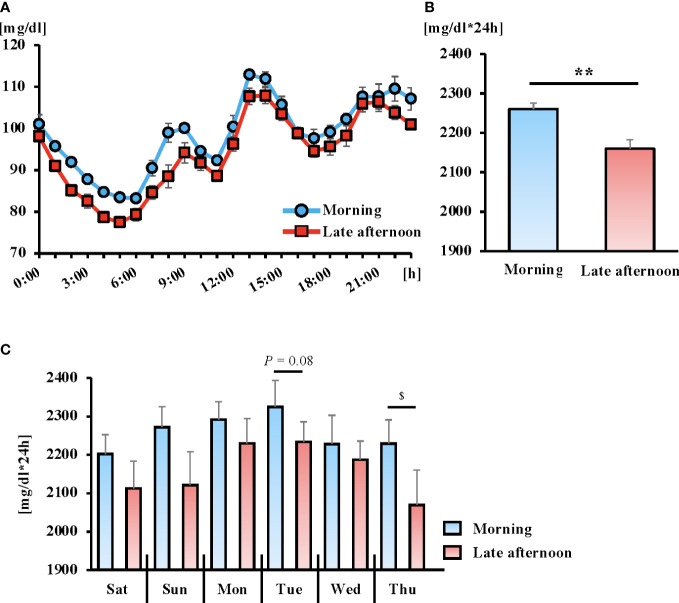
The 6-day average of the 24-hour blood glucose concentrations **(A)** and area under the curve (AUC) **(B)** following the exercise intervention. Daily AUC for 6 days following the exercise intervention **(C)**. Blue and red lines indicate the morning and late afternoon trials, respectively. All data are presented as mean ± standard error. **P < 0.01 compared to the level in the Morning trial (paired t-test). ^$^ *P* < 0.05 compared to the level in the Morning trial (Wilcoxon). Blood glucose fluctuations were calculated from the 24-hour blood glucose fluctuations after the intervention for each trial (from Saturday to next Thursday). The 24-h glucose variability measurements were taken from 00:00 h to 24:00 h on that day.

There were no significant differences in 24-h glucose variability during the intervention period in either trial (**Figure S2A**). The AUC of blood glucose variability during the intervention period was lower in the late afternoon trial; however, the difference was not statistically significant (**Figure S2B**). The daily AUC for each trial did not differ significantly between trials (**Figure S3**). On the other hand, in the 24-h blood glucose variation after the intervention of each trial, the late afternoon trial showed lower values than the morning trial (trial, *P* = 0.002; time, *P* < 0.001; interaction, *P* = 0.571), and the AUC was significantly lower (*P* < 0.01) (**Figures 3A, B**). In addition, the daily AUC after the intervention for each trial was lower in the late afternoon versus morning trial, with significantly lower Thursday AUC values (**Figure 3C**; *P* < 0.05).

### Comparison of postprandial glucose concentrations and AUC

**Figure 4:**
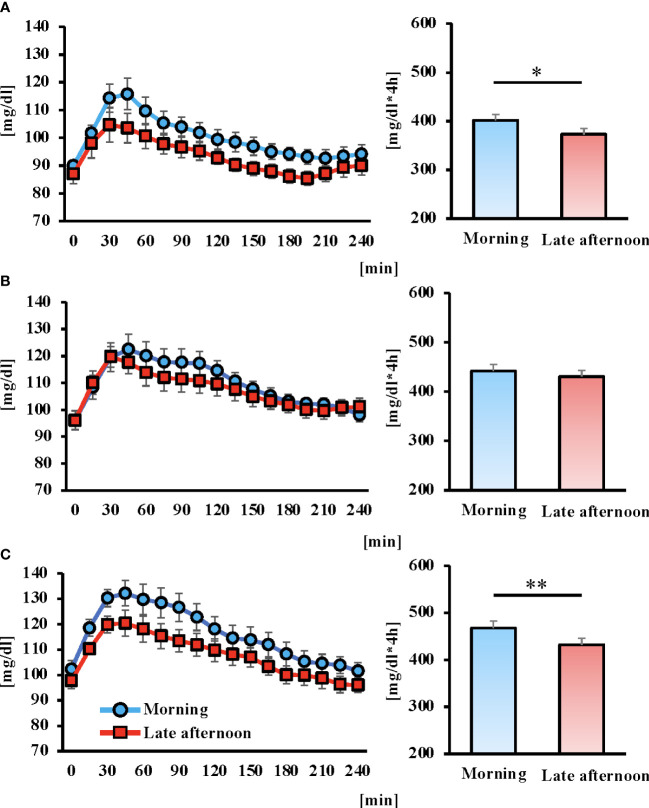
Concentrations of glucose of postprandial 4 hours and area under the curve (AUC) for 6 days after intervention in each trial. Breakfast **(A)**, lunch **(B)** Dinner **(C)**, and the blue and red lines indicate the morning and late afternoon trials, respectively. All data are presented as mean ± standard error. *P < 0.05, **P < 0.01 compared to the level in the Morning trial (paired t-test).

Postprandial blood glucose fluctuations were lower in the late afternoon versus morning trial for all three meals. The AUC after breakfast and dinner was significantly lower in the late afternoon versus morning trial (**Figure 4**; *P* < 0.05, *P* < 0.01, respectively).

### Comparison of glucose parameters between trials

The mean, minimum, and J-index values were significantly lower in the late afternoon versus morning trial (*P* < 0.01), whereas there were no significant differences in MAX and MAGE between trials (**Table 2**).

**Table 2:** Comparison of glucose parameters between the morning and evening trials.

	Morning trial	Late afternoon trial
**Mean (mg/dl)**	98.5 ± 0.7	94.1 ± 1.0**
**MAX (mg/dl)**	115.9 ± 2.2	113.0 ± 1.6
**MIN (mg/dl)**	82.7 ± 0.6	76.9 ± 1.0**
**MAGE (mg/dl)**	33.1 ± 2.4	36.0 ± 2.0
**J-index**	11.7 ± 0.3	10.9 ± 0.2**

All data are presented as mean ± standard error, SD: standard deviation, MAX: maximum glucose, MIN: minimum glucose, MAGE: mean amplitude of glycaemic excursion. **P < 0.01 compared to the level in the Morning trial (paired t-test). Glucose parameters were calculated from the 24-hour blood glucose fluctuations after the intervention for each trial (from Saturday to next Thursday). The 24-h glucose variability measurements were taken from 00:00 h to 24:00 h on that day.

### Blood lipids and parameters before versus after intervention by trial

**Figure 5:**
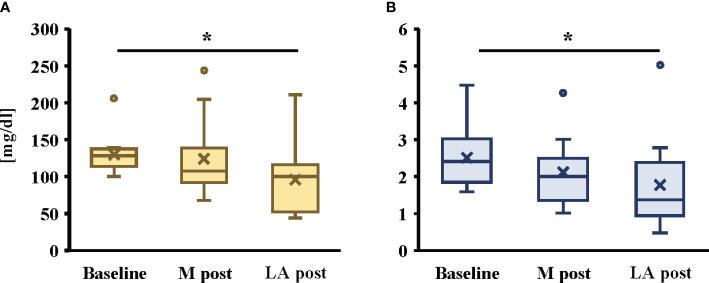
Changes in TG **(A)** and TG/HDL-C ratio **(B)** by morning or late afternoon of short-term endurance exercise. All data are presented as mean ± standard error. *P < 0.05 compared to the level in the Morning trial (Friedman). TG: triglycerides, HDL-C: high-density lipoprotein cholesterol, M post: After the intervention of morning trial, LA post: After the intervention of late afternoon trial. The participants were required to abstain from any intense exercise for at least one day before the collection of the blood sample and fast for at least 10 h overnight. The blood samples were collected at 09:00–10:00, and blood samples after each trial intervention were drawn at least 48 h after the end of the last trial.

A significant decrease in TG and TG/HDL-C ratio after the intervention was noted in the late afternoon trial only (**Figure 5**; *P* < 0.05, both). However, there were no significant changes in glucose, insulin, HDL-C, LDL-C, ApoA1, ApoB, ApoB/Apo A1, or CUC in either trial (**Table 3**).

**Table 3:** Comparison of blood parameters.

	Baseline	M post	LA post
**Glucose (mg/dl)**	88.5 ± 5.0	86.1 ± 6.1	96.1 ± 6.0
**Insulin (μU/mL)**	24.0 ± 4.7	19.1 ± 2.8	22.1 ± 5.6
**TG (mg/dl)**	129.8 ± 7.9	123.9 ± 15.1	96.2 ± 13.5
**HDL-C (mg/dl)**	53.9 ± 3.1	60.0 ± 3.8	60.9 ± 4.9
**LDL-C (mg/dl)**	89.9 ± 6.7	87.4 ± 6.8	86.1 ± 6.2
**ApoA1 (mg/dl)**	142.2 ± 6.9	150.3 ± 8.4	146.9 ± 8.1
**ApoB (mg/dl)**	69.9 ± 3.9	67.3 ± 4.3	67.5 ± 4.5
**ApoB/ApoA1**	0.51 ± 0.04	0.47 ± 0.04	0.48 ± 0.05
**CUC (unit/ml)**	99.9 ± 6.2	116.7 ± 11.5	113.1 ± 10.6

All data are presented as mean ± standard error, TG: triglycerides, HDL-C: high-density lipoprotein cholesterol, LDL-C: low-density lipoprotein cholesterol, ApoA1: apolipoprotein A1, ApoB: apolipoprotein B, CUC: cholesterol uptake capacity, M post: After the intervention of morning trial, LA post: After the intervention of late afternoon trial. Venous blood samples were collected at baseline and after each trial. The participants were required to abstain from any intense exercise for at least one day before the collection of the blood sample and fast for at least 10 h overnight. The blood samples were collected at 09:00–10:00, and blood samples after each trial intervention were drawn at least 48 h after the end of the last trial.

## Discussion

The present study examined the effects of short-term morning or late afternoon endurance exercise on diurnal variations in blood glucose and lipid levels in healthy young men. The diurnal variation in blood glucose levels after the exercise intervention was lower in the late afternoon versus morning trial. Blood glucose fluctuations using AUC showed significantly lower values in the late afternoon versus morning trial. The AUC of blood glucose fluctuation after each meal also showed lower values in the late afternoon than morning trial for all three meals and significantly lower values in the late afternoon versus morning trial for breakfast and dinner. In addition, only the late afternoon trial showed significant reductions in TG and TG/HDL-C levels after versus before the intervention. These results suggest that late afternoon endurance exercise may be more effective than morning endurance exercise at improving 24-h glucose and triglyceride levels.

The insulin-sensitizing effect of exercise is explained by the AMPK-mediated translocation of GLUT4 to the subsarcolemmal membrane, thereby promoting insulin-stimulated glucose uptake into muscle cells (29). Indeed, previous studies in humans reported that the interaction between exercise-induced AMPK phosphorylation and the subsequent insulin-stimulated activation of GLUT4 alleviates 24-h hyperglycemia (30, 31). In contrast, glucose tolerance exhibits diurnal variation and is reportedly lower in the evening versus morning (32). In addition, insulin sensitivity and beta cell responsiveness to glucose, which are thought to contribute to decreased glucose tolerance, are lower at dinner than at breakfast (33, 34). Thus, temporal variations in insulin sensitivity and the time dependence of the insulin secretion rate are thought to contribute to the diurnal variation in glucose metabolism. Therefore, the effects of exercise on blood glucose fluctuations at different times of day may differ. In the present study, diurnal fluctuations in blood glucose levels were lower in the late afternoon versus morning trial. A previous study of patients with type 2 diabetes reported that afternoon high-intensity interval training improved glycemic profiles more effectively than morning training (35). These findings are consistent with data from mice showing that glycolytic activation is more specific to exercise during the early active phase than during the early rest phase (36). Therefore, these results show that glycemic control during endurance exercise is more effective in the late afternoon versus morning.

The mechanism that promotes glucose initiatives in exercise has been shown to involve myokines, which are secreted by muscle contraction during exercise. IL-6, one of the myokines, has been shown to have multifunctional properties with important roles in regulating immune and inflammatory responses and is secreted in a manner dependent on exercise intensity and duration of exercise (37). Furthermore, IL-6 has been shown to be involved in glucose metabolism, facilitating the uptake of blood glucose into muscle cells; IL-6 induces GLUT4 translocation onto the plasma membrane by activating AMPK in muscle cells (38). The resulting translocation of GLUT4 receptors to the muscle cell membrane promotes blood glucose uptake and lowers blood glucose levels. The previous study has shown that changes in blood IL-6 concentrations after acute endurance exercise are higher in the evening than in the morning (22). Therefore, differences in the change in IL-6 concentrations after morning or late afternoon endurance exercise may have influenced the results. Another possibility is its association with insulin secretory function. However, blood insulin levels in the morning or late afternoon endurance exercise were not significantly different between trials. Therefore, the effect of insulin secretory function is considered to be small. On the other hand, previous studies have shown that short-term endurance exercise increases glucagon-like peptide-1 (GLP-1) and improves beta-cell function (39). Furthermore, a previous study has shown that the endocrine function of incretin extends beyond its stimulatory effect on insulin secretion, and GLP-1/glucose-dependent insulinotropic polypeptide (GIP) receptors are also found in skeletal muscle, a cell outside the pancreas (40). In other words, incretins have been shown to regulate glucose uptake into muscle in an insulin-independent manner. Therefore, late afternoon endurance exercise may have affected diurnal blood glucose fluctuations by improving beta cells and increasing incretin secretion. However, the present study did not include myokine or incretin, and more detailed studies are needed in the future.

The effect of glycemic control on short-term endurance exercise lasts for up to a week. Muscle sensitivity to insulin stimulation increases over several hours (up to 48 h) after exercise (41). In addition to the effects of acute exercise, insulin activity is reportedly enhanced over a longer period with repeated exercise (41). The 24-h blood glucose variability after the endurance exercise intervention in this study was lower in the late afternoon than morning trial. Furthermore, the effect lasted for approximately 1 week. Phosphatidylinositol 3-kinase (PI 3-kinase) is an important step in the regulation of glucose transport, and its activation is required for insulin-mediated glucose transport in insulin-sensitive tissues such as skeletal muscle (42, 43). Indeed, PI 3-kinase plays a specific role in insulin-stimulated glucose transport (42, 43). Short-term exercise training reportedly enhances insulin-stimulated PI 3-kinase activity in humans and rodents (44, 45). These findings suggest that enhanced insulin signaling may contribute to the increased insulin action of exercise in human skeletal muscles. Thus, insulin action may be more enhanced after late afternoon versus morning endurance exercise.

TG and cholesterol synthesis show diurnal variations in which their levels are higher at night (46, 47). In other words, lipid tolerance is lower in the evening than in the morning. In fact, evening statin intake more effectively improves cholesterol levels (48). Therefore, the effect of exercise on blood lipid levels may vary according to time of day. TG level is an independent risk factor for CVDs. Epidemiological studies have shown a higher prevalence of dyslipidemia in patients with versus those without CVDs, indicating an association between elevated serum TG levels and an increased risk of CVDs (49, 50). In the present study, TG levels decreased significantly after versus before the intervention only in the late afternoon endurance exercise trial. Previous studies demonstrated that exercise improves the risk of CVD, including dyslipidemia and hyperglycemia (51, 52). Another previous study examined the effects of different exercise timings over 12 weeks on blood lipids and inflammatory markers in 330 patients with cardiovascular disease. The results showed more improvement in LDL-C with evening exercise compared to morning exercise (53). This indicates that the effect of exercise on blood lipid levels may vary according to time of day.

Dyslipidemia is a major risk factor for CVDs, and exercise is a beneficial way to improve blood lipid levels (54). The decrease in TG levels observed in this study may be due to lipoprotein lipase (LPL). In a previous study, exercise reportedly increased LPL mRNA expression and decreased blood TG levels (55). Furthermore, LPL activity in response to a meal load was lower in the evening than in the morning (56). Therefore, the effect of late afternoon exercise on TG levels may have been more pronounced by promoting LPL activity. However, the contribution of LPL to reducing blood TG levels obtained in this study has not been examined and requires further investigation.

No differences in HDL-C or LDL-C levels were found in morning versus late afternoon endurance exercise. A previous study demonstrated that exercise can improve HDL-C levels (57). On the other hand, a sufficient training volume is necessary to improve blood cholesterol levels, while total energy expenditure and intensity are important factors related to improvement (52). The short-term endurance exercise focused on in this study may have been insufficient to cause changes in blood cholesterol because its type and intensity differed from those in the previous study. Furthermore, the diurnal values of TG fluctuate by 33–63%, whereas LDL fluctuates by less than 10%, and HDL-C shows no or very small diurnal fluctuations (18, 20, 58). ApoA1 and ApoB also have diurnal fluctuations of 24 and 12 h, respectively, but their diurnal fluctuations are small (5–6%) (18). Therefore, their effects on morning or late afternoon endurance exercise may be small.

This study has several limitations. First, it included only healthy young men. In a previous study, men were shown to have up to twice as high postprandial TG concentrations and greater diurnal variation than women (59). These sex-based differences are thought to be due to the effects of estrogen on lipid metabolism (60). Therefore, differences in the time of day of exercise implementation to TG may show different results according to sex. Second, a previous study has shown similar diurnal variation in glucose tolerance in patients with type 2 diabetes between morning and evening (61). A previous study in overweight with type 2 diabetes and pre-diabetic subjects showed similar effects on glycemic control during a 12-week morning and evening exercise intervention (62). Therefore, different subjects may have different effects on glycemic variability at different times of day during exercise. Third, the time of exercise intervention used in this study differs from previous studies (22, 23, 62). Hormones and other factors involved in energy metabolism have been shown to have diurnal variations (22, 63). Therefore, the effects on glucose fluctuations and blood lipids may differ depending on the time of day when exercise is performed. Finally, phosphorylation of the AMPK subunit complexes during exercise exhibits diurnal variation and is highly dependent on exercise intensity and type (64, 65). Hence, it is necessary to examine the effects of exercise on blood glucose fluctuations at different exercise times and consider exercise intensity and type.

## Conclusion

In conclusion, our study findings demonstrated that short-term evening endurance exercise was more effective than morning endurance exercise at controlling blood glucose levels during the day, an effect that lasted for approximately 1 week. Evening exercise was more effective at improving blood lipid levels than morning exercise.

## Data availability statement

The original contributions presented in this study are included in the article/**Supplementary Material**, and further inquiries can be directed to the corresponding author.

## Ethics statement

The studies involving human participants were reviewed and approved by Ethics Committee for Humans at Waseda University. The patients/participants provided their written informed consent to participate in this study.

## Author contributions

The authors’ responsibilities were as follows: H-KK, SF, and SS, conceptualization; SF and H-KK, data curation; H-KK, SF, and MT, formal analysis; H-KK and SS, funding acquisition; H-KK, SF, HC, TN, and MT, investigation; H-KK, MT, and SS, study methodology; H-KK and SS, project administration; SS and H-KK, project resources; SS, supervision; H-KK and SF, visualization; H-KK, SF, and SS, writing—original draft; MT, TI, ZR, and SZS, writing, review, and editing. All authors have read and approved the final manuscript for publication.

## Funding

This work was supported by the Japan Society for the Promotion of Science (KAKENHI grant numbers 20K19689 and 18K17940) to H-KK and 19H01089 to SS), and the JST-Mirai Program (JMPJM120D5 for SS), Japan. The funder was not involved in the study design; data collection, analysis, and interpretation; writing of this article; or the decision to submit the article for publication.

## Conflict of interest

The authors declare that the research was conducted in the absence of any commercial or financial relationships that could be construed as a potential conflict of interest.

## Publisher’s note

All claims expressed in this article are solely those of the authors and do not necessarily represent those of their affiliated organizations, or those of the publisher, the editors and the reviewers. Any product that may be evaluated in this article, or claim that may be made by its manufacturer, is not guaranteed or endorsed by the publisher.
