# Supplementary material for: Late-afternoon endurance exercise is more effective than morning endurance exercise at improving 24-h glucose and blood lipid levels
Source: Front Endocrinol (Lausanne). 2022 Jul 19;13:957239. doi: 10.3389/fendo.2022.957239 (PMC9343590; doi:10.3389/fendo.2022.957239)
Supplement: Supplementary file 1 [file DataSheet_1.docx]

Supplementary Material

# Supplementary Data

Supplementary Material should be uploaded separately on submission. Please include any supplementary data, figures and/or tables. All supplementary files are deposited to FigShare for permanent storage and receive a DOI.

Supplementary material is not typeset so please ensure that all information is clearly presented, the appropriate caption is included in the file and not in the manuscript, and that the style conforms to the rest of the article. To avoid discrepancies between the published article and the supplementary material, please do not add the title, author list, affiliations or correspondence in the supplementary files.

# Supplementary Figures and Tables

For more information on Supplementary Material and for details on the different file types accepted, please see [here](http://home.frontiersin.org/about/author-guidelines#SupplementaryMaterial). Figures, tables, and images will be published under a Creative Commons CC-BY licence and permission must be obtained for use of copyrighted material from other sources (including re-published/adapted/modified/partial figures and images from the internet). It is the responsibility of the authors to acquire the licenses, to follow any citation instructions requested by third-party rights holders, and cover any supplementary charges.

## Supplementary Figures

**
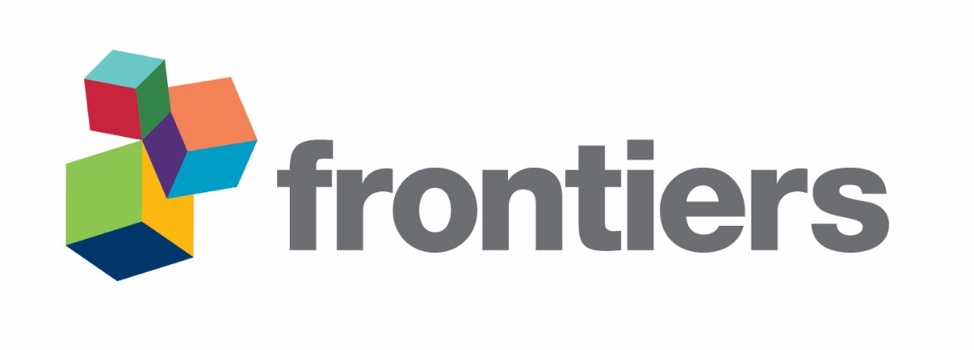
**

**Supplementary Figure 1.** The figure legends are required to have the same font as the main text, 12 point normal Times New Roman, single spaced. Please use a single paragraph for each legend and prepare the figures keeping in mind the PDF layout.

**Supplementary Table 1.** Physical activity levels and meal-time in both trials period.

|  | **Morning trial period** | **Late afternoon trial period** |
| --- | --- | --- |
| **Physical activity** |  |  |
| **Step count (steps/day)** | 8492.6 ± 1210.4 | 9601.9 ± 1266.9 |
| **MVPA (min/day)** | 110.9 ± 7.2 | 118.5 ± 8.4 |
| **Meal time** |  |  |
| **Breakfast time (h:min)** | 8:16 ± 0:07 | 8:26 ± 0:07 |
| **Lunch time (h:min)** | 12:45 ± 0:04 | 12:48 ± 0:06 |
| **Dinner time (h:min)** | 19:59 ± 0:11 | 20:00 ± 0:07 |

All data are presented as mean ± standard error. MVPA: moderate-to-vigorous physical activity.

**Supplementary Table 2.** Energy intake before and after the entire study period.

|  | **Baseline** | **After intervention** |
| --- | --- | --- |
| **Energy intake (kcal/day)** | 2100.2 ± 102.3 | 2041.9 ± 93.3 |
| **Protein (g/day)** | 80.3 ± 4.1 | 75.6 ± 3.3 |
| **Fat (g/day)** | 70.2 ± 5.0 | 74.5 ± 5.7 |
| **Carbohydrate (g/day)** | 266.2 ± 15.0 | 250.0 ± 8.5 |

All data are presented as mean ± standard error.

**Supplementary figure 1.** Comparison of the heart rate during the exercise in each trial. Blue and red bars indicate the morning and late afternoon trials, respectively. All data are presented as mean ± standard error. (A) Monday trial, (B) Wednesday trial, and (C) Friday trial.

**Supplementary Figure 2.** Diurnal changes in the glucose concentration during the intervention period (A) and area under the curve (B) in both trials. Blue and red lines indicate the morning and late afternoon trials, respectively. All data are presented as mean ± standard error.

**Supplementary Figure 3.** Comparison of the area under the curve for the intervention period in both trials. Blue and red bars indicate the morning and late afternoon trials, respectively. All data are presented as mean ± standard error. * Blood glucose fluctuations were also measured on Monday, but the values for the first day were not stable, so they were excluded from the analysis.
